# Heterogeneity in Meta-Analyses of Genome-Wide Association Investigations

**DOI:** 10.1371/journal.pone.0000841

**Published:** 2007-09-05

**Authors:** John P.A. Ioannidis, Nikolaos A. Patsopoulos, Evangelos Evangelou

**Affiliations:** 1 Clinical and Molecular Epidemiology Unit, Department of Hygiene and Epidemiology, University of Ioannina School of Medicine, Ioannina, Greece; 2 Biomedical Research Institute, Foundation for Research and Technology-Hellas, Ioannina, Greece; 3 Department of Medicine, Tufts University School of Medicine, Boston, Massachusetts, United States of America; University of Michigan, United States of America

## Abstract

**Background:**

Meta-analysis is the systematic and quantitative synthesis of effect sizes and the exploration of their diversity across different studies. Meta-analyses are increasingly applied to synthesize data from genome-wide association (GWA) studies and from other teams that try to replicate the genetic variants that emerge from such investigations. Between-study heterogeneity is important to document and may point to interesting leads.

**Methodology/Principal Findings:**

To exemplify these issues, we used data from three GWA studies on type 2 diabetes and their replication efforts where meta-analyses of all data using fixed effects methods (not incorporating between-study heterogeneity) have already been published. We considered 11 polymorphisms that at least one of the three teams has suggested as susceptibility loci for type 2 diabetes. The I^2^ inconsistency metric (measuring the amount of heterogeneity not due to chance) was different from 0 (no detectable heterogeneity) for 6 of the 11 genetic variants; inconsistency was moderate to very large (I^2^ = 32–77%) for 5 of them. For these 5 polymorphisms, random effects calculations incorporating between-study heterogeneity revealed more conservative p-values for the summary effects compared with the fixed effects calculations. These 5 associations were perused in detail to highlight potential explanations for between-study heterogeneity. These include identification of a marker for a correlated phenotype (e.g. *FTO* rs8050136 being associated with type 2 diabetes through its effect on obesity); differential linkage disequilibrium across studies of the identified genetic markers with the respective culprit polymorphisms (e.g., possibly the case for *CDKAL1* polymorphisms or for rs9300039 and markers in linkage disequilibrium, as shown by additional studies); and potential bias. Results were largely similar, when we treated the discovery and replication data from each GWA investigation as separate studies.

**Significance:**

Between-study heterogeneity is useful to document in the synthesis of data from GWA investigations and can offer valuable insights for further clarification of gene-disease associations.

## Introduction

Meta-analysis entails the combination of different studies or datasets on the same research question and meta-analytic methods have been used across many different scientific disciplines [1], [2]. Early applications of meta-analysis in the 1970s and 1980s proposed that a major gain from these methods was the ability to improve power and obtain more definitive summary results by combining several small studies [3]. However, it soon became evident that simply focusing on summary effects could be misleading. For epidemiological applications in particular, a major threat is that the precision derived from combining data may be spurious, especially if the combined studies and datasets have considerable dissimilarities [4]. It is well appreciated now that besides estimating summary effects, estimation and, if possible, explanation, of the between-study heterogeneity is a very important goal for meta-analysis [5].

One of the most rapidly growing applications of meta-analysis is in genetic epidemiology [6]–[8]. Meta-analysis is becoming standard practice for publications of genome-wide association studies that search for common genetic variants regulating complex traits and disease risk. A torrent of such studies have started appearing in the most prestigious journals with major prospects for the delineation of the genetic risk factors underlying the most common diseases and traits [9]. The results of the genome-wide associations are typically combined with the results of additional replication studies on the most promising variants; occasionally results from other genome-wide investigations are also included in meta-analytic calculations [10]–[13]. However, these early applications of meta-analyses on such datasets have not accommodated between-study heterogeneity in the data synthesis. In the presence of between-study heterogeneity in the genetic effects, there may be important implications for the interpretation of the results.

We exemplify this issue for meta-analyses of genome-wide association and replication data on postulated genetic variants conferring susceptibility to type 2 diabetes [10]–[13]. We have revisited these data to examine the extent of between-study inconsistency, whether summary results may differ with consideration of between-study heterogeneity, and what insights may be gleaned from the presence of between-study heterogeneity in this setting.

## Methods

### Fixed versus random effects

Data were combined in the original *Science* publications [10]–[13] using a fixed effects (Mantel-Haenszel) model. Fixed effects assume that the genetic effects are the same across the combined investigations and all differences are due to chance [1], [2]. While this assumption is true occasionally, it may not be generalizable to all genetic associations. Genetic effects may vary across different populations for various reasons, including both genuine differences and differential biases and errors across studies [14], [15]. In meta-analyses, fixed effects may give more narrow confidence intervals and more impressively low p-values compared with models that accommodate potential diversity of effects (heterogeneity) [1], [2], [5], [16], [17]. We have re-analyzed the meta-analyses of the three teams with random effects calculations [1], [2]. Random effects calculations assume that due to genuine differences and or different biases, the estimates of the genetic effects may vary across different investigations. Random effects thus try to estimate the population average and the extent of dispersion in these different effect sizes. The presented random effects calculations use the DerSimonian and Laird estimator of the between-study variance [18].

### Heterogeneity metrics

Different metrics have been proposed for testing the presence and measuring the amount of between-study heterogeneity. *Cochran's Q statistic* [19] is provided by *Q* = Σ *w_i_^F^* (*d_i_*−*d^F^*_+_)^2^ where *d^F^*_+_ is the summary effect size by fixed effects, *d_i_* are the study-specific effect sizes and *w_i_^F^* is the weight of each study (based on Mantel-Haenszel methods). The statistic follows a *x* ^2^ distribution with *k*-1 degrees of freedom (k is the number of studies or datasets combined), and it is typically considered significant at the α = 0.10 level. The original *Science* publications used this test to document whether there is or not between-study heterogeneity. However, this test is grossly underpowered, when there are very few studies. Also with small studies, the confidence intervals of each one may be very large, so the same problem of lack of power may still persist. Of note, Q is used in the estimation of the between-study variance, given by 
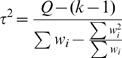
. The ratio of *τ* over the effect size conveys the extent of variability (between-study standard deviation) as compared with the effect size.

Another useful metric is the *I^2^*. This metric is independent of the number of studies and can be compared across meta-analyses with different number of studies and metrics [16], [17]. I^2^ is given by the formula 
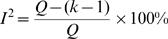
 and it is a measure of the percentage of total variation across studies due to heterogeneity beyond chance. Therefore, I^2 ^takes values between 0–100%. Values over 50% indicate large heterogeneity. I^2 ^can be estimated along with its confidence intervals and the confidence intervals are wider when a meta-analysis includes few studies [20]. The confidence intervals for I^2^ can be calculated with different methods (described in detail in [17]). Confidence intervals usually can be very large, unless many studies are available, and this is another indication that one has to be cautious about claiming homogeneity (even when I^2^ is zero). Overall, there can be large uncertainty in a meta-analysis about the presence or not and the extent of between-study heterogeneity. Strong inferences about heterogeneity or lack thereof may be a common misconception when limited data are available.

### Datasets

Data are derived from the original *Science* publications of 3 GWA investigations and their replication efforts (advance online publications in *Science* on April 26, 2007 [10], [11], [13]). These investigations were conducted by the Finland-United States Investigation on NIDDM Genetics (FUSION) team, the Diabetes Genetics Initiative, and the Wellcome Trust Case-Control Consortium. Extensive details of the design and populations of these investigations have been presented in the original publications [10], [11], [13]. In brief, all three publications used a discovery dataset where GWA evaluation was performed on Illumina or Affymetrix chips and promising genetic variants were further tested for replication in large replication datasets. The number of polymorphisms tested in the discovery phase of each investigation were 317503, 500568, and 499032, respectively and the number of polymorphisms that were considered to have data suitable for analyses were 315635, 386731, and 393453, respectively. The FUSION investigation used 1161 cases and 1174 controls in the discovery phase and 1215 cases and 1258 controls in the replication phase. The Diabetes Genetics Initiative investigation used 1464 cases and 1467 controls in the discovery phase and 5065 cases and 5785 controls in the replication phase. The Wellcome Trust Case control Consortium used 1924 cases and 2938 controls in the discovery phase and 3757 cases and 5346 controls in the replication phase.

In order to identify which of the many promising polymorphisms were eventually most important as susceptibility loci for type 2 diabetes, the three investigations performed a meta-analysis of their data. All three publications eventually reported tables of “confirmed” susceptibility loci of type 2 diabetes. We examined all 11 genetic variants that have been listed in these tables as “confirmed” loci. Eight of those variants appear in the “confirmed” lists of all 3 publications; while rs9300039 is listed in the table of confirmed loci by FUSION investigators only [10]; rs564398 (a second marker in the *CDKN2B* gene besides the rs10811661 that is considered confirmed by all three publications) is listed in the table of confirmed loci by the Wellcome Trust Case Control Consortium investigators only [13]; and *FTO* rs8050136 (an obesity risk variant, as discussed below) is listed in the table of confirmed loci by the Wellcome Trust Case Control Consortium and FUSION only [10], [13]. These differences reflect simply minor differences in interpretation and listing of the same results between the three teams of investigators.

We treated each GWA analysis and its replication efforts as one study, as in the original *Science* publications. Therefore, three studies were analyzed for between-study heterogeneity and meta-analysis was performed with random effects on these 3 estimates, as described above. In a sensitivity analysis, for each GWA investigation, we treated the GWA discovery data as a separate study from the GWA replication data. Thus in the sensitivity analysis, between-study heterogeneity was estimated and random effects meta-analysis was performed considering a maximum 6 estimates. Some markers had not been pursued for testing in the replication phase of all 3 GWA investigations, thus they are represented by 5 or 4 estimates in the sensitivity meta-analysis. We should caution that even with this further split, each of the estimates may still be composed on several sub-studies. For example, the replication efforts may be comprised on many smaller teams and their data have already been synthesized (again using fixed effects assumptions), but separate data for these sub-studies are not consistently available. In some cases, the pieces would be even impossible to separate, as for example when the group of cases was composed of many smaller samples recruited from different places, while the control group was more uniformly recruited. By accepting that the sub-studies are sufficiently homogeneous, the estimates of between-study heterogeneity that we present may tend to be even underestimates of the full heterogeneity that may exist in the data.

For each of the polymorphisms where we identified moderate or larger estimates of between-study heterogeneity (I^2^≥25%), we discuss the potential insight that may be offered by this heterogeneity and how it may affect or not the interpretation of the results. We also retrieved additional studies that have been published on the same or linked polymorphisms as of May 12, 2007 on type 2 diabetes or related phenotypes so as to consider potentially additional evidence from other relevant studies in the interpretation of the results. Additional studies were identified by PubMed searches using “diabetes” and “association” limited to the year 2007. Relevant additional data that were available were also incorporated into updated random effects meta-analyses.

### Software

All calculations have been performed in Intercooled STATA 8.2 (College Station, TX) using the *metan* module. The presented confidence intervals for I^2^ are obtained using the non-central chi-square distribution-based method [17] using the *heterogi* STATA module. P-values are two-tailed.

## Results

### Main analyses

As shown (Table 1), the I^2^ metric was different from 0 (no detectable heterogeneity) for 6 of the 11 genetic variants. Inconsistency of the genetic effects across the three investigations was very large (I^2^≥75%) for rs9300039 and *FTO* rs8050136, moderate (I^2^ between 25–50%) for *PPARG* rs1801282, *CDKAL1* rs10946398 and *SLC30A8* rs13266634, and low (I^2^ up to 25%) for *IGF2BP2* rs4402960. In fact the upper 95% CI of I^2^ extended up to very high levels of inconsistency (73–91%) for all 11 polymorphisms; thus, between-study heterogeneity in the genetic effects cannot be confidently excluded for any of them. Conversely, even when the I^2^ estimate is high, the 95% confidence intervals typically do not exclude the possibility of homogeneity. I^2^ is an indicator, not absolute proof of homo- or heterogeneity.

**Table 1:** Between-study heterogeneity and random versus fixed effects calculations for polymorphisms that were considered “confirmed”

GENE	Polymorphism	Q (p)	I^2^ (95% CI)	Random effects OR (95% CI)	Fixed effects OR (95% CI)	Random effects p-value	Fixed effects p-value
*—*	rs9300039^a^	7.98 (0.019)	75% (0–90)	1.25 (1.04–1.50)	1.25 (1.15–1.37)	0.015	4.3×10*-7*
*FTO*	rs8050136	8.62 (0.013)	77% (0–91)	1.13 (1.02–1.25)	1.17 (1.12–1.22)	0.015	1.3×10^−12^
*PPARG*	rs1801282	3.80 (0.15)	47% (0–84)	1.16 (1.07–1.25)	1.14 (1.08–1.20)	0.0003	1.7×10^−6^
*CDKAL1*	rs10946398^b^	3.73 (0.16)	46% (0–84)	1.12 (1.07–1.17)	1.12 (1.08–1.16)	3.2×10^−6^	4.1×10^−11^
*SLC30A8*	rs13266634	2.92 (0.23)	32% (0–81)	1.12 (1.07–1.18)	1.12 (1.07–1.16)	8.7×10^−6^	5.3×10^−8^
*CDKN2B*	rs564398	1.48 (0.48)	0% (0–73)	1.12 (1.07–1.17)	1.12 (1.07–1.17)	1.2×10^−7^	1.2×10^−7^
*HHEX*	rs5015480–rs1111875	0.45 (0.80)	0% (0–73)	1.13 (1.08–1.17)	1.13 (1.08–1.17)	5.7×10^−10^	5.7×10^−10^
*KCNJ11*	rs5215^c^	0.56 (0.76)	0% (0–73)	1.14 (1.10–1.19)	1.14 (1.10–1.19)	5×10^−11^	5×10^−11^
*IGF2BP2*	rs4402960	2.65 (0.27)	25% (0–79)	1.15 (1.10–1.19)	1.14 (1.10–1.18)	6.5×10^−12^	8.6×10^−16^
*CDKN2B*	rs10811661	0.03 (0.99)	0% (0–73)	1.20 (1.14–1.25)	1.20 (1.14–1.25)	7.8×10^−15^	7.8×10^−15^
*TCF7L2*	rs7901695^d^	0.24 (0.89)	0% (0–73)	1.37 (1.31–1.43)	1.37 (1.31–1.43)	1.0×10^−48^	1.0×10^−48^

Additive models are presented, as in the main analyses of the original papers. Fixed effects calculations are Mantel-Haenszel estimates as in the original papers. Random effects calculations use the DerSimonian and Laird estimators for the between-study variance.

CI: confidence interval; OR: odds ratio

a multi-marker tag in DGI and rs1514823 in the UK study

b rs7754840 in FUSION

c rs5219 in FUSION and DGI

d rs7903146 in FUSION and DGI

The heterogeneity test (Cochran's Q statistic) was formally statistically significant for the *FTO* variant (p = 0.014) and the rs9300039 marker (p = 0.018). As Q is grossly underpowered with only 3 studies, lack of nominal statistical significance for heterogeneity in the other polymorphisms does not prove homogeneity of effects. Of interest, the two loci with highest-between study heterogeneity were not unanimously proposed as “conformed” susceptibility loci by all 3 GWA investigations.

The summary point estimates (odds ratios) are practically not different with random versus fixed effects with 8 of the 11 pairs of estimates being identical to the second decimal point (Table 1). However, for the 5 variants with moderate to very large between-study heterogeneity, the 95% confidence intervals expand substantially with random effects calculations and p-values do not satisfy criteria for genome-wide significance (p<10^−7^) based on these data alone.

### Sensitivity analyses

Sensitivity analyses considering the discovery and replication data of each GWA investigation as separate data yielded largely similar results as the main analysis (Table 2). Minor differences were seen in the exact estimates of I^2^ but the categorization of the amount of between-study heterogeneity was largely similar. With this analysis, there was now also low heterogeneity (I^2^<25%) for the *HHEX* rs5015480-rs1111875 effect, while I^2 ^became 0 for *SLC30A8* rs13266634. Again the confidence intervals of I^2^ were very large, the upper limit being between 61 and 86% for the various markers.

**Table 2:** Between-study heterogeneity and random versus fixed effects calculations for polymorphisms that were considered “confirmed” in sensitivity analyses considering the discovery and replication data of each GWA as a separate study.

GENE	Polymorphism	Q (df)^a^ [p]	I^2^ (95% CI)	Random effects OR (95% CI)	Fixed effectsOR (95% CI)	Random effectsp-value	Fixed effectsp-value
*—*	rs9300039	8.38 (3) [0.039]	64% (0–86)	1.29 (1.11–1.50)	1.26 (1.15–1.37)	0.001	2.8×10^−8^
*FTO*	rs8050136	12.98 (4) [0.011]	69% (0–86)	1.15 (1.06–1.25)	1.17 (1.12–1.23)	0.001	2.5×10^−12^
*PPARG*	rs1801282	6.93 (4) [0.14)	42% (0–76)	1.14 (1.06–1.23)	1.13 (1.08–1.20)	0.0007	3.4×10^−6^
*CDKAL1*	rs10946398	8.76 (5) [0.12]	43% (0–76)	1.13 (1.07–1.18)	1.12 (1.08–1.15)	1.2×10^−6^	1.9×10^−10^
*SLC30A8*	rs13266634	3.17 (5) [0.67]	0 (0–61)	1.13 (1.08–1.17)	1.13 (1.08–1.17)	4.1×10^−9^	4.1×10^−9^
*CDKN2B*	rs564398	3.62 (4) [0.46]	0% (0–64)	1.11 (1.06–1.15)	1.11 (1.06–1.15)	5.8×10^−7^	5.8×10^−7^
*HHEX*	rs5015480–rs1111875	6.20 (5) [0.29]	19% (0–68)	1.13 (1.08–1.17)	1.12 (1.08–1.17)	2.2×10^−8^	3.2×10^−10^
*KCNJ11*	rs5215	3.50 (4) [0.48]	0% (0–64)	1.14 (1.09–1.18)	1.14 (1.09–1.18)	9×10^−11^	9×10^−11^
*IGF2BP2*	rs4402960	7.08 (5) [0.21]	29% (0–71)	1.15 (1.10–1.20)	1.15 (1.11–1.19)	2.9×10^−10^	1.1×10^−15^
*CDKN2B*	rs10811661	4.15 (5) [0.53]	0% (0–61)	1.20 (1.15–1.25)	1.20 (1.15–1.25)	2.7×10^−15^	2.7×10^−15^
*TCF7L2*	rs7901695	1.31 (4) [0.86]	0% (0–64)	1.37 (1.32–1.43)	1.37 (1.32–1.43)	1.0×10^−48^	1.0×10^−48^

CI: confidence interval; OR: odds ratio

a df = degrees of freedom; not all markers were tested by all 3 investigations in their replication efforts, thus even with splitting the discovery and replication phases, there are fewer than 6 datasets (df = 5) for some variants.

### Examination of markers with moderate or larger between-study heterogeneity

Examination of the 5 polymorphisms that had moderate or larger between-study heterogeneity in their effect sizes shows that detection of between-study heterogeneity can offer useful insights.

The weakest evidence for association was seen for rs9300039 that was listed by the FUSION investigators as a confirmed susceptibility locus for type 2 diabetes [10]. The random effects summary odds ratio yielded a mere p = 0.015, as compared with p = 4.3×10^−7^ by Mantel-Haenszel calculations, and there was very large between-study heterogeneity (75%). Heterogeneity might reflect in part the different tag polymorphisms used in the other two GWA investigations [11], [13]. Even so, the evidence remains very weak. Fine mapping and more extensive data would be required for this locus before a concrete claim can be made that it confers susceptibility to type 2 diabetes.

**Figure 1:**
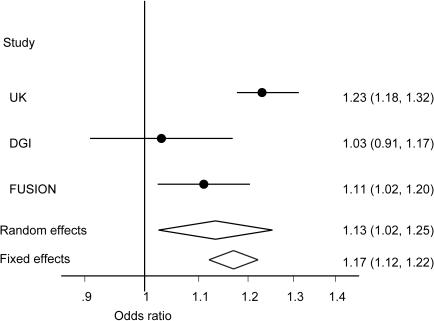
Meta-analysis of the *FTO* rs8050136 variant in terms of its association with type 2 diabetes across three GWA investigations. Each investigation is shown by the point estimate of the odds ratio and 95% confidence intervals. Also shown is the diamond of the summary effect by fixed and random effects calculations.

For *FTO* rs8050136, the random effects summary odds ratio yielded also a mere p = 0.015, as compared with the impressive Mantel-Haenszel p = 1.3×10^−12^ originally reported. Between-study heterogeneity is also very large (77%). Heterogeneity is visible even with plain data inspection, especially for the Wellcome Trust Case Control Consortium vs. Diabetes Genetics Initiative results (figure 1). Consistent with this strong signal of between-study heterogeneity, the Wellcome Trust investigators have indeed found that this variant is a susceptibility marker for increased body mass index and obesity [12]. Type 2 diabetes susceptibility may be mediated through the effect on body mass index and is not an independent effect that should have been seen consistently in all populations. The observed heterogeneity for type 2 diabetes association is also explained by the study design of the 3 GWA investigations. The Diabetes Genetics Initiative used a tightly matched case-control sample in the discovery phase, where cases and controls had been matched for body mass index [11] and thus it is not surprising that there was no residual effect of this *FTO* variant on the risk of type 2 diabetes.

For the novel proposed *CDKAL1* rs10946398 association, additional data were recently published from deCODE and affiliated investigators in *Nature Genetics* [21] who proposed a different polymorphism in the same gene (rs7756992) as a T2D marker. The r^2^ for these two markers was only 0.67 in Caucasians, but 4 other *CDKLA1* polymorphisms in the deCODE data [21] have r^2^ = 1 with rs10946398. Including the deCODE data for the nearest one (rs7774594) in the meta-analysis calculations, the summary odds ratio became 1.13 (95% CI, 1.08–1.18) with p = 2.2×10^−7^. Moderate between-study heterogeneity persisted (I^2^ = 43%). In the deCODE presented results for rs7756992 (ref. 13), we estimated very large between-population heterogeneity in the genetic effects between three different racial descent populations (I^2^ = 76%). Compared with Caucasians, the correlation between rs10946398 (or rs7774594) and rs7756992 was much weaker in Africans (r^2^ = 0.35). In Africans, deCODE investigators noticed that rs7756992 showed no association effect (odds ratio 1.02). The very large heterogeneity in genetic effects for rs7756992 and the moderate heterogeneity for rs10946398 might suggest that neither polymorphism is the true culprit; the culprit may be more consistently correlated (even in Africans) with rs10946398 than with rs7756992.

For *SLC30A8* rs13266634 data were also available from the deCODE investigation [21] and from another study by Sladek et al. [22]. Including these data, we got a random effects odds ratio of 1.15 (95% 1.10–1.19) with p = 3.5×10^−13^. Some low heterogeneity persisted (I^2^ = 21%). While *SLC30A8* rs13266634 amply passed genome-wide significance when additional data were considered, the presence of between-study heterogeneity suggests the true culprit variant may still be elusive.

The same may apply to *PPARG* rs1801282, which had been extensively studied in the past (before the GWA investigations) in many other studies, again with clear association in the summary effect, but with some co-existing between-study heterogeneity [23]. Efforts to identify the true functional culprit variants of *PPARG* (if different from rs1801282) are ongoing. By comparison, the other two variants that been already known and extensively studied before the 3 GWA investigations (*KCNJ11* rs5215 and *TCF7L2* rs7901695) have I^2^ = 0 in both the main and sensitivity meta-analyses.

## Discussion

In a re-analysis of the data from 3 GWA studies on type 2 diabetes, we found that for 5 of the 11 genetic variants that are considered “confirmed” susceptibility loci for type 2 diabetes there was still moderate to very large between-study heterogeneity across the different GWA investigations. Given the between-study heterogeneity, the level of statistical significance was more conservative with random effects calculations. Further examination of these potentially heterogeneous associations suggested possible explanations for the observed inconsistency. In several cases, this probably reflected either the fact that the identified marker was not the culprit polymorphism, but had a different linkage disequilibrium pattern with the culprit polymorphism across different studies. In the case of *FTO*, it probably reflected the fact it was associated with type 2 diabetes through its effect on the correlated phenotype of obesity; the phenotype correlation varied across different studies. Additional possibilities may need to be considered also for the heterogeneity, as discussed below. Conversely, we should caution that homogeneity of effects for the other 6 variants provides limited information on whether a causative locus has been identified. Lack of heterogeneity is not proof of causality.

Overall, detection of heterogeneity is very useful. Some polymorphisms are shown to reach genome-wide statistical significance by fixed effects calculations, but not by random effects calculations due to large between-study heterogeneity. In these cases, priority should be given to the consideration of other, correlated phenotypes and fine mapping for identifying linked, true culprit polymorphisms that yield less heterogeneous association signals. These situations are likely to be very common in the GWA setting. Tag markers are not selected based on “candidate gene variant” considerations [24]. Thus it is more likely that one may hit upon a variant that is a linked marker rather than hit directly upon the culprit causative variant. Markers will often have variable linkage disequilibrium across different populations. This will result in heterogeneous genetic effects across studies.

Correlated phenotypes are also a major issue. Many common diseases and traits (e.g. diabetes, myocardial infarction, obesity, hypertension, metabolic syndrome) are modestly or even highly correlated. Inconsistent susceptibility signals for one of them may reflect consistent associations with another correlated phenotype. Moreover, most common diseases that are assumed to have a complex genetic background are probably a complex mix of different phenotypes in terms of their molecular pathogenesis. Genetic variants may have specific molecular functional effects that cumulatively build a complex clinical phenotype. However, depending on their molecular background, the relative representation of these phenotypes may vary in different people and populations with seemingly the same clinical disease. The case definition of this broad clinical phenotype may not do justice to the underlying molecular complexity. Molecular and clinical phenotypes may exhibit some correlation pattern, but this may vary in different sub-populations depending on the presence of other gene variants. Again, statistical heterogeneity may offer a window to this complexity.

Another possibility is bias. Incorporating between-study heterogeneity in the summary calculations has the advantage to penalize associations where results are inconsistent across studies due to population-specific biases and gives higher ranks to the consistent associations [25]. The 3 GWA investigations on type 2 diabetes paid meticulous attention to methodological detail and their design was exemplary. Careful genotyping controls were set and population stratification was controlled with principal component analysis [26]. Nevertheless, minute biases affecting particular polymorphisms with minute odds ratios around 1.12 cannot be excluded. Even if some major systematic errors (e.g. population stratification, genotyping error, phenotype misclassification) are controlled, not all biases are foreseeable. Moreover, minimized average biases do not exclude much larger differential biases for a few polymorphisms. P-values for testing the observed genetic effects against the null effect hypothesis account for random, not systematic, error.

Another potential reason for heterogeneity is the winner's curse, a manifestation of chance and regression-to-the-mean, especially under circumstances of multiple testing with limited power. The first study that claims an association that passes a very demanding required significance threshold may exhibit a genetic effect that is larger than the true average effect of this association.

Finally, another possibility is gene-environment interactions (e.g. as proposed for rs1801282 and low physical activity [27]) with differential non-genetic environmental exposures across different populations. Moreover, genuine genetic heterogeneity in effect sizes across different ethnic backgrounds and population-specific gene-gene epistatic effects are sometimes postulated. However, interaction effects (effect modification) may require huge studies to confirm [28], much larger than even the very large consortia that have been put together in the genetics of type 2 diabetes.

We should stress that estimation of between-study heterogeneity carries considerable uncertainty and in the typical situation it would be impossible to have a large number of large studies to fully power detection and accurate estimation of heterogeneity. Moreover, breaking down populations to sub-studies may sometimes lead to loss of estimated between-study heterogeneity, if the sub-studies are small and their confidence intervals of effects are very large. However, this would offer misleading reassurance that no heterogeneity exists. While the number of datasets may increase by such splitting, each dataset would have very limited, inconclusive information about the magnitude of the effect and it would again be very difficult to show the between-study heterogeneity, even if present.

In general, when between-study heterogeneity is demonstrated or cannot be excluded, random effects models have been accepted as the default across different applications of meta-analysis and this should be accepted also for GWA investigations [1], [2], [5]. Fixed effects may sometimes result into misleading inferences. In the presence of heterogeneity, the main assumption of fixed effects is violated and their application is inappropriate. However, a caveat for random effects is that they tend to diminish the difference in the relative weighting of small vs. larger studies. This is a drawback in situations where small studies may suffer more from errors or biases than larger studies. Disproportionate weighting of the biased small studies would then lead to erroneous results. This situation may typically arise when the data to be synthesized have been collected retrospectively from published information and publication bias is operating in the field [27]. Small studies may have been published preferentially when they show significant results while the evidence from larger studies may be available regardless of the results. Thus the total available evidence from larger studies may be more unbiased, even if single larger studies may not necessarily be more unbiased than single smaller studies.

While this bias is a concern for retrospective meta-analyses, it should not be an issue for a prospective collaborative GWA investigation performed within a consortium of investigators. In this setting, there is no reason why investigators would select to include in the calculations only the most impressive results. However, a particular threat for the credibility of GWA results occurs, if several GWA investigations are performed and results are made available only for the most significant p-values in each GWA investigation. While this deficit will hopefully be remedied by quick release of genome-wide data in the future, the majority of studies have not done so yet.

We should also mention that there are different models that can incorporate between-study heterogeneity in the calculations. We used a conservative approach, the DerSimonian and Laird model, that is the most frequently used random effects model in the literature. Other fully Bayesian approaches may also be used [29], including hierarchical Bayesian models. Some of these models may incorporate also other parameters such as minor deviations from Hardy-Weinberg equilibrium in the observed genotyping data [30]. These models usually tend to give even larger uncertainty and they widen the 95% credibility intervals of the estimates [29].

In all, heterogeneity is a useful aspect of the data, rather than a nuisance, as it can often point to leads that can clarify better the nature of postulated associations in the context of meta-analysis [31]. Heterogeneity should not be ignored and should be carefully factored in the interpretation of emerging genetic associations from GWA studies. Heterogeneity has implications also for the epidemiological design of GWA studies and their replication efforts. Consistency in the definition of phenotypes and meticulous attention to quality control in genotyping and avoidance of population stratification is warranted, so as to avoid heterogeneity due to bias. However, heterogeneity due to genuine differences should not be avoided. Thus one should encourage diversity in secondary aspects of the study design across studies, such as the use of matching or not for other population characteristics, and targeting of populations of diverse racial descent with different linkage disequilibrium patterns. Finally, proper evaluation of between-study heterogeneity would ideally require complete and transparent individual-level information on genotype results from all conducted GWA investigations. Ensuring full public data availability would enhance the credibility of GWA evidence.
